# Recent Trends in Morphology-Controlled Synthesis and Application of Mesoporous Silica Nanoparticles

**DOI:** 10.3390/nano10112122

**Published:** 2020-10-25

**Authors:** Nabanita Pal, Jun-Hyeok Lee, Eun-Bum Cho

**Affiliations:** 1 Department of Physics and Chemistry, Mahatma Gandhi Institute of Technology, Gandipet, Hyderabad 500075, India; nabanitapal_chem@mgit.ac.in; 2 Department of Fine Chemistry, Seoul National University of Science and Technology, Seoul 01811, Korea; ljhfive@seoultech.ac.kr

**Keywords:** silica nanoparticles, morphology, applications, sensing, drug delivery

## Abstract

The outstanding journey towards the investigation of mesoporous materials commences with the discovery of high surface area porous silica materials, named MCM-41 (Mobil Composition of Matter-41) according to the inventors’ name Mobile scientists in the United States. Based on a self-assembled supramolecular templating mechanism, the synthesis of mesoporous silica has extended to wide varieties of silica categories along with versatile applications of all these types in many fields. These silica families have some extraordinary structural features, like highly tunable nanoscale sized pore diameter, good Brunauer–Emmett–Teller (BET) surface areas, good flexibility to accommodate different organic and inorganic functional groups, metals etc., onto their surface. As a consequence, thousands of scientists and researchers throughout the world have reported numerous silica materials in the form of published articles, communication, reviews, etc. Beside this, attention is also given to the morphology-oriented synthesis of silica nanoparticles and their significant effects on the emerging fields of study like catalysis, energy applications, sensing, environmental, and biomedical research. This review highlights a consolidated overview of those morphology-based mesoporous silica particles, emphasizing their syntheses and potential role in many promising fields of research.

## 1. Introduction

In recent time, porous silica is one of the most significant developments in the nanotechnology world. Highly porous silica materials are thermally stable, chemically inert, inexpensive, easy to synthesize, non-toxic, biocompatible, and they also have very good flexibility towards surface functionalization [1]. Consequently, experts throughout the world have paid much attention to employ this porous silica in multiple fields like catalysis [2], sensing [3], energy application [4], optically active materials [5], drug delivery [6], etc.

Although, the preparation of mesoporous silica was mentioned in some US patents around the year 1970 [7], it was not so popular among the researchers until 1990 [8]. In 1992, Kresge et al., from Mobil Corporation laboratories, produced a new family of mesoporous materials, called MCMs, or “Mobil Composition of Matters” [9]. Another well-known silica family, Santa Barbara Amorphous (SBA) which includes hexagonal and large pore silica SBA-15 have been synthesized by Stucky et al. [10]. The synthesis of almost all types of silica materials is based on the self-assembly of inorganic precursors and organic structure directing agents (SDA) under acidic or alkaline conditions [9,11]. Additionally, an accurate monitoring of the synthesis conditions, like surfactants, silica precursors, solvents or cosolvents, pH of the media, variation of reaction, or hydrothermal temperature can result the generation of mesoporous silica nanoparticles with different morphologies, variable surface area, pore size, as well as orientation of the pore channels [12]. Recently, a simple non-polar solvent-assisted Stöber method has been reported by Wang and his co-workers to synthesize ordered mesoporous silica particles with diverse morphologies and mesostructures [13]. Similarly, periodic mesoporous silicas (PMOs) with hollow spherical structure have been developed by Teng et al., while using various organic moieties [14]. These morphology-controlled syntheses of silica nanoparticles are quite popular now-a-days, due to the potential role of these materials in multiple areas of research [2,6].

In last few years, a huge number of publications that are based on the significant applications of various morphology-oriented silica nanoparticles have come to light. However, a combined overview in the form of review article is also required in order to help the budding scientists obtain precise information about this area of research. To the best of our knowledge, a proper review article on the wide range of applications of various morphology-based silica nanoparticles has not been reported to date. Hence, in this work we have focused on the synthesis of different porous silica materials having interesting morphologies and their pivotal role in many applied fields. 

## 2. Synthesis Pathway for Morphology-Controlled Mesoporous Silica Nanoparticles

**Figure 1:**
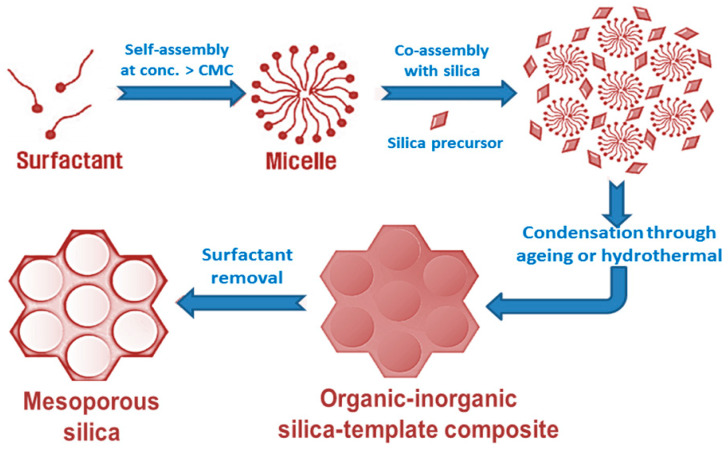
General synthesis strategy for mesoporous silica using a surfactant-templated route.

According to International Union of Pure and Applied Chemistry (IUPAC) classification, mesoporous solids, with a pore diameter in the range of 2–50 nm, is one of the main categories of ‘nanoporous’ materials, which are designed as a continuous solid framework material having voids or ‘nanoscale pores’ of the order of say 100 nm or smaller [11]. Most common methodology for synthesis of mesoporous silica is by using quaternary alkylammonium surfactants (e.g., cetyltrimethylammonium bromide, CTAB) as a template, under highly basic conditions or utilizing triblock copolymers like, Pluronic F127, P123, etc., in strong acidic conditions [9,15,16]. Though there is some variation in the synthesis conditions and structural properties of porous silicas, but the basic strategy of all the syntheses is based on the supramolecular self-assembly of the surfactants (or templates). In the solution phase, co-operative self-assembly of organic surfactant molecules takes place at concentrations higher than CMC (critical miceller concentration) and in alkaline or acidic conditions, hydrolyzed inorganic silica precursors (say, tetraethyl orthosilicate or TEOS) arrange themselves around these template micelles in order to form organic–inorganic silica-template composites [11]. The further condensation of metal and template occurs through proper ageing for definite time and the composites take an ordered arrangement such as cubic or hexagonal, etc., silica with mesopores with particular diameter is obtained after the removal of the organic surfactant molecules from the composite materials through calcination or solvent-extraction [17]. Figure 1 shows a detailed synthesis strategy for mesoporous silica using a surfactant-templated route.

Surfactant self-assembly and the related factors like surfactant types, its concentration, counter ions, pH of the media etc., which affect the self-assembly process, have a very crucial impact on the size and shapes of the formed mesopores [11]. For e.g., CTAB surfactant self-assembly under highly basic condition can generate a highly ordered hexagonal pore in mesoporous silica, whereas its self-assembly in highly acidic results in the formation of cubic mesopores [18]. In the same fashion, the structure, shapes or size of porous nanoparticles is highly dependent on the types, concentration of the surfactant and silica precursors, their concentration, counter ions or other ions present, temperature of the reaction, pH of the medium, solvent, etc. [9]. Consequently, porous nanoparticles with different morphologies are formed. The imperative effects of these morphology oriented nanoporous particles on various applied fields is one of the main issues of the recent research world. Different morphologies of silica nanoparticles and their applications will be emphasized in this review.

**Figure 2:**
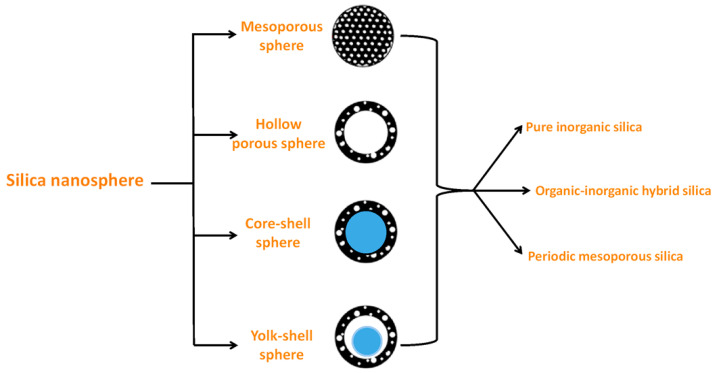
General synthesis strategy for mesoporous silica using a surfactant-templated route.

Porous silica, organic functionalized or metal-doped silica particles can have varying morphologies, depending on the synthesis conditions or the reagents that were used for synthesis. In the following sections, the various morphologies of silica nanoparticles (Figure 2) will be systematically elaborated. 

### 2.1. Porous Silica Nanosphere

Among all of the morphologies, reports on spherical type silica and organosilica nanoparticles with the nanoscale level porosity are most abundant in literature. Silica particles can be found with pores the range of micropore (<2 nm) to mesopore (2–50 nm) arranged in an ordered and non-ordered way [1]. In 1956, Kolbe et al., first synthesized highly dispersed non-porous silica particles with the help of silicon alkoxide hydrolysis and condensation principle in alcohol [19]. Later, the controlled synthesis of micrometer-sized monodispersed silica sphere has been mentioned by Stöber et al., in 1968 [20]. They used ammonia as base as well as morphology-controlling catalyst to produce spherical particles by the hydrolysis of alkyl silicate in alcoholic medium. For surfactant-free synthesis of mesoporous silica nanosphere with tunable pore diameter (100–230 nm) role of ammonia has been proved to be very crucial. Here, a facile two step pathway by premixing three components TEOS-H_2_O-EtOH with definite molar ratios followed by addition of ammonia, then the treatment of the resulting silica by water dilution and soft-etching with ammonia produce monodispersed porous silica sphere [21]. Usually, mesostructured spherical silica material, like MCM-41 with hexagonally ordered mesopores, can be produced based on the condensation of self-assembled cationic surfactant and hydrolysed silica precursor, tetraethyl orthosilicate or TEOS under highly alkaline condition [11]. Additionally, instead of TEOS, use of commercially available silica gel say, Lichrosphere 100 results pseudomorphic synthesis of MCM-41 with perfect spherical shape [22]. In the same way, pseudomorphic synthesis of large size spherical shaped Co-doped MCM-41 has been reported by Lim et al., who synthesized the material using commercial silica, cobalt salt and CTAB surfactant in highly basic medium [23]. Presence of Na ion in the reaction medium inhibits the homogeneous distribution of Co species in the silica matrices. Thus, well-defined spherical morphology of Co-MCM-41 was achieved by optimizing the autoclaving time to four days. On the other hand, in HCl acidic media long ageing time has also been employed by Alonso’s group to synthesize functionalized silica sphere using CTAB, TEOS, and isopropanol based on spray-drying method [24]. 

**Figure 3:**
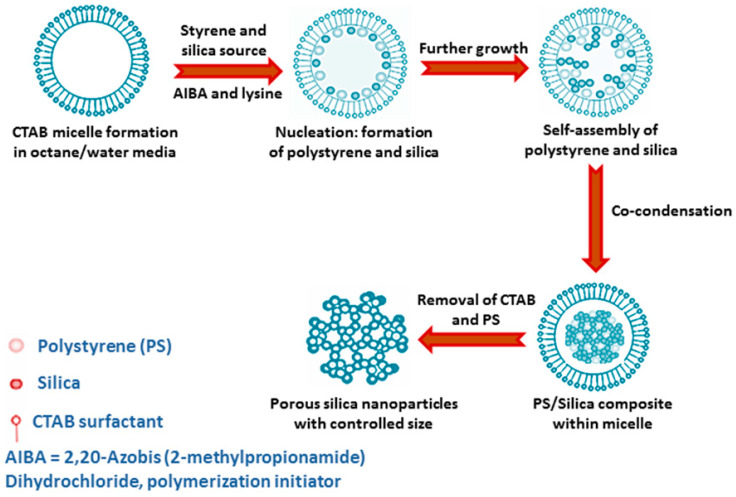
A schematic representation for mesoporous silica nanoparticles formation in presence of Cetyltrimethylammonium bromide-Polystyrene (CTAB-PS) and amino acid lysine.

Sometimes, by using weakly basic amino acid like arginine the hydrolysis of TEOS can be controlled at moderate pH (9–10), which results in the formation of discrete silica nanosphere of 20–40 nm size [25]. Moreover, the size of the particles can be varied significantly by changing the stirring rate, amount of water, or cationic surfactant C_16_TMACl. Surfactant cetyltrimethylammonium chloride (C_16_TMACl) has also been used by Qiao et al., in order to synthesize uniform mesoporous spherical silica sphere at moderate pH = 6–10 in the presence of certain suitable additives like diethanol amine, triethanol amine, ammonia or any inorganic salts [26]. Monodispersed silica nanospheres can also be obtained simply using sodium acetate additive in the presence of C_16_TMACl surfactant and TEOS at moderate reaction temperature ca. 60 °C without adding any other alkalis [27]. In order to control the particle growth in nanometre range, the use of amino acid like lysine has been mentioned by Nandiyanto and his group. Here, the synthesis of mesoporous spherical silica sphere of 20–80 nm diameter was done using styrene and CTAB surfactant in water-octane media [28]. Styrene is polymerized in the reaction medium in order to produce polystyrene that acts as a template for this silica preparation, because a comparative study showed that, with increasing styrene concentration, particle size and pore size both gradually increase. Figure 3 shows a schematic representation for synthesis of mesoporous silica nanoparticles in the presence of CTAB-PS and amino acid lysine. 

In 2010, Polshettiwar et al., mentioned the formation of fibrous silica nanospheres while using cationic template like cetylpyridinium bromide (CPB) or CTAB and urea as hydrolysing agent in a solvent mixture of cylcohexane, pentanol, and water [29]. The role of non-polar template chain cetyl group and the polar head pyridinium or trimethylammonium group to produce this well-defined fibrous morphology is very crucial, owing to the surfactant packing parameter. Not only that, fabrication of this fibrous nanosilica sphere is highly dependent on precise control of urea amount as well as proper selection of combined solvent, cyclohexane, and pentanol. It was also observed that the resulting silica spheres have very high mechanical and thermal stability. 

While using a neutral template like octylamine or dodecylamine, uniform monodispersed spherical silica and thiol (SH-)-functionalized silica can be obtained in highly acidic solution by using a water-ethanol solvent mixture, as revealed by Kosuge et al. [30]. Additionally, when compared to octylamine, dodecylamine has been proved to be more effective structure-directing agent to produce ordered mesoporous silica and organosilica nanospheres with higher morphological quality. In HCl medium, Coulombic interaction between charged species, say long-chain protonated amine (S^+^), coordinating anions of Cl- (X^−^), and positively charged silicate oligomer (I^+^), facilitates the S^+^X^−^I^+^ type mechanism, which favours the formation of this silica and functionalized silica hard spheres. Similar mechanism was observed in case of hard sphere Al-doped porous silica synthesis [31]. Another organosilica, say rhodamine spirolacturm derivative functionalized mesoporous silica synthesized using CTAB-NaOH mixture in aqueous media, has also produced uniform spherical type particles of 75–85 nm dimension [32]. Spherical mesoporous silica particles with diameter of 65–740 nm have been prepared by Nooney et al., by using a varying quantity of the initial reagents and changing template from cationic to neutral or reaction media from homogeneous to heterogeneous [33]. 

The formation of monodispersed silica nanosphere while using Pluronic triblock copolymer templates and sodium silicate in strong acidic aqueous media is highly dependent on the parameter like presence of counterions viz. Cl^−^ and NO_3_^−^, which control the hydrophilicity of the reaction media resulting precise spherical shape [34]. Additionally, the particle size has been monitored by changing the reactants ratio, stirring speed, or by mixing of surfactants. Pluronic template F127 can also act as particle dispersion agent to synthesize monodispersed cubic mesostructured MCM-48 silica nanosphere in a CTAB templated basic water-ethanol media [35]. A diluted solution of CTAB and higher concentration of F127 facilitates the formation of well-defined smaller sized MCM-48 nanoparticles. In the similar way, a combination of surfactants CTAB and Brij-56 has been employed by He et al., in order to synthesize highly dispersed ordered mesoporous spherical silica nanoparticles under buffer maintained neutral pH condition [36]. Here also, the role of non-ionic surfactant Brij-56 is to improve the morphology and dispersivity of the silica nanoparticles, although the mesostructure was little disrupted by this. Monodispersed silica microsphere can be obtained by the evaporation induced CTAB-Brij 58 templating method while using vibrating orifice aerosol generator, as reported by Rama Rao et al. [37].

Use of a dispersing agent to synthesize well-dispersed three-dimensional (3D) porous silica sphere has also been reported by Fu and his co-workers [38]. They used carboxymethyl cellulose as dispersing agent in a dodecyltrimethylammonium bromide surfactant-mediated to fabricate cubic silica nanoparticles of ca. 60 nm via the rapid two-step pH-modulated method. Huo et al., reported the preparation of porous hard sphere silica particles using tetrabutyl orthosilicate (TBOS) as silica source in aqueous NaOH media [39]. Here, the desired morphology of silica is obtained while hydrophobic TBOS and butyl alcohol that was produced from the hydrolysis of TBOS in basic media creates oil-in-water emulsion and stabilized by the quaternary ammonium CTAB surfactant. Han et al. reported another strategy using mixed surfactants. They used triblock copolymer surfactant (e.g., F127, P123) and cationic fluorocarbon surfactant FC-4 for the preparation of well-dispersed mesoporous silica nanosphere in weakly acidic media [40]. The role of FC-4 is to get ultrafine particles by controlling the growth of particle, while mesostructure formation is facilitated by copolymer template. 

Highly dispersed spherical shaped colloidal mesoporous silica (CMS) functionalized with different organic groups, like vinyl-, benzyl-, phenyl-, cyano-, mercapto-, aminopropyl-, etc., can be synthesized in a combined media of triethanolamine (TEA) and C_16_TMACl using mixture of TEOS and a variety of organoalkoxysilanes [41]. In the same way, colloidal silica nanoparticles with spherical morphology have also been reported by Yamada et al., who showed that by using different alkoxysilane (Si(OR)_4_, R = CH_3_, C_2_H_5_, C_3_H_7_, C_4_H_9_), four types of mesoporous silica nanospheres with different diameters (20–80 nm) can be prepared due to different hydrolysis rate of the silica precursors [42]. Another monodispersed colloidal suspension of porous silica spheres of very small dimension (15–30 nm) have been synthesized while using CTAB surfactant, triethanolamine catalyst, silica precursors TEOS, and phenyl functionalized silica, followed by extraction using HCl-ethanol and ammonium nitrate-ethanol [43]. A homogeneous thin film was also successfully prepared using this small nanoparticle suspension. In 2016, Curcumin based fluorescent organic-inorganic hybrid colloidal PMO is prepared by Datz et al., with the help of a mixture of Curcumin functionalized silica and bis(triethoxysilyl)ethane (BTEE) without TEOS in CTAB mediated alkaline water-ethanol media [44].

**Figure 4:**
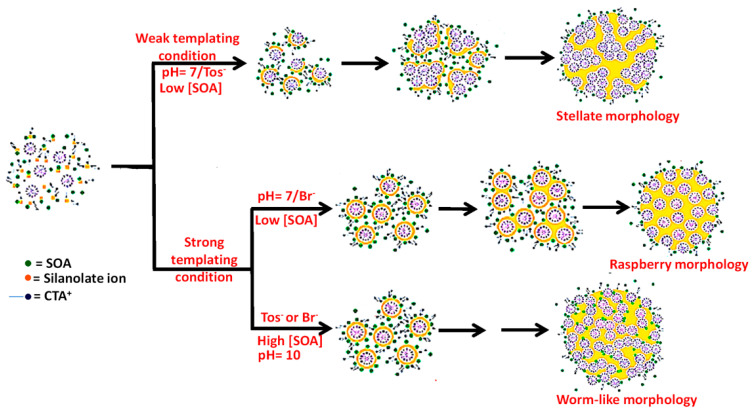
Schematic representations of pathways of silica-template organic-inorganic composite nanostructures formation under various conditions.

Zhang et al. reported the large scale production of well-dispersed porous silica sphere [45]. They showed that the fabrication of nanospherical silica with <130 nm particle size can be done by while using an appropriate cationic surfactant, cetyltrimethylammonium (CTA^+^) with suitable counterions (Tosylate, Tos^−^, or bromide, Br^−^) in the presence of small organic amines (SOAs), including triethyleneamine, triethanolamine, and 2-amino-2-(hydroxymethyl)propane-1,3-diol as minerali- zing agents. Meso-channelled spherical morphology, including stellate, raspberry, and worm-like structure, is controlled by adjusting proper SOA concentration and counterion types. The method results in the development of ‘weak templating condition’ (low silanolate density at pH = 7 due to high concentration of Tos^−^ around CTA^+^ micelle) and ‘strong templating condition’ (high SOA concentration or presence of Br^−^ ions and high silanolate density), which also enables high yield and large-scale production of mesoporous silica nanospheres. Figure 4 elaborates the formation pathways of silica-template organic-inorganic composite nanostructures. Yu et al. prepared uniform spherical silica nanospheres with dendritic pore channels, using imidazolium ionic liquids (ILs) with different alkyl lengths as cosurfactants in presence of triethanolamine, cetyltrimethylammonium tosylate, and TEOS [46].

Surface functionalization of silica materials has been demonstrated by Bagwe et al. in order to reduce nanoparticle aggregation [47]. Surface modification was carried out using TEOS and different organosilanes in aqueous ammonia media based on a water-in-oil (W/O) or reverse microemulsion method. It was observed that the surface modification of silica with amine groups is further supported by an inert group (e.g., methyl phosphonate), for which highly monodispersed silica nanospheres have been obtained. Cheng et al. also reported well-dispersed mesoporous silica nanoparticles by proper functionalization [48]. 

Modified Stöber method has been employed for synthesis of well-defined spherical and monodispersed PMO by Rebbin and co-workers [49]. They used different long-chain tetraalkylammonium halide as surfactants and 1,2-bis(trimethoxysilyl)ethane (BTME) as silica precursors in ammoniacal water-ethanol media in order to obtain this PMO. Using the similar method, another PMO nanosphere having ethylene groups in their framework has been synthesized by Xia et al., in the presence of bis(triethoxysilyl)ethylene (BTEE) precursor and CTAB template [50]. 

### 2.2. Mesoporous Silica Hollow Sphere

**Figure 5:**
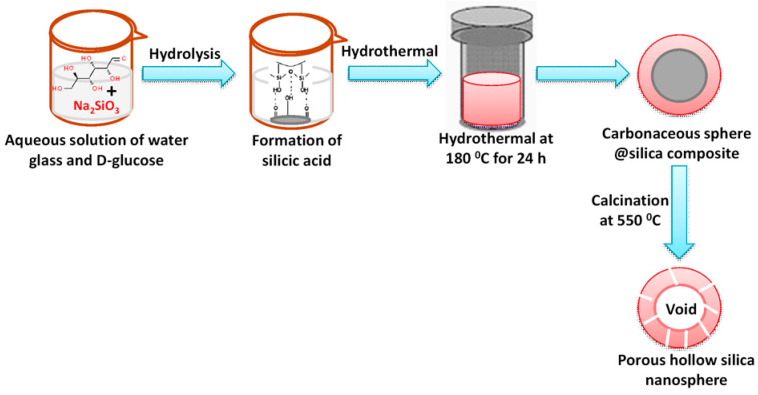
A simple strategy for synthesis of porous silica hollow sphere.

The difference between a sphere and a hollow sphere is that the hollow sphere has another internal sphere inside it, whereas normal sphere is entirely solid. Hollow sphere silica nanoparticles are gaining more importance owing to their indispensable role in biomedical applications, catalysis and so on [14,51,52]. The synthesis of hollow silica sphere generally follows dual-templating pathway [1]. The outer shell is porous whereas the inner shell is solid made from organic polymers or metal oxides. Bruinsma et al. reported the preparation of hollow silica sphere via a spray drying method based on surfactant C_16_TMACl, TEOS, and water, in the very early stage of mesoporous synthesis [53]. Recently, more advanced aerosol-assisted spray-drying method has been proposed by Poostforooshan et al., to synthesize porous hollow silica sphere [54]. An aqueous suspension of colloidal silica nanoparticles and template Eudragit/Triton X100 composite have been used in different molar ratio in order to produce hollow silica microsphere with tunable pore mesoporous shell. A simple one-pot hydrothermal route for synthesis of hollow silica sphere is also mentioned by Abdelaal et al., in the year 2014 [55]. Water glass or sodium silicate has been used as silica precursor and D-glucose as sacrificial template. In acidic media, a spherical nanocomposite has been formed having hydrolysed glucose as core and silica as shell, then calcination in air removes the inside carbonaceous core leaving the space vacant. It was observed that the morphology, shell thickness, as well as the particle size of the hollow silica sphere are highly dependent on the amount of water glass precursor and concentration of glucose template. Moreover, the cross-linking of silicic acid during thermal treatment generates porosity in the silica shell [55]. Figure 5 shows a simple strategy for synthesis of this porous silica hollow sphere.

Cationic surfactant CTAB has been proved a wonderful substance to assist in the formation of hollow mesoporous silica nanosphere [56]. The process was made successful with the fabrication of solid silica nanosphere that was based on modified Stöber method, then the adsorption of CTAB on this silica sphere followed by Na_2_CO_3_ etching, subsequent redeposition, and etching process results in the transformation of the solid sphere into hollow mesoporous silica sphere [56]. The role of CTAB is most important here as it acts as soft template to form mesoporous shell, as promoter to speed up the etching process of solid silica sphere, as well as a stabilizer to shield the silicate-CTAB shell during alkaline etching process. The hollow interior of the sphere and thickness and stability of the shell, etc., can be nicely tuned by varying the concentration of TEOS during solid silica sphere preparation or CTAB amount, respectively [56]. Surfactant-directing alkaline etching process using Na_2_CO_3_ is also observed during the preparation of hollow silica nanocapsules that were reported by Chen et al. [57]. The pore size of the silica shell can be adjusted between 3 to 10 nm by a reversible Si-O bond breaking and reforming process, whereas completely nonporous hollow nanocapsules can be prepared by regrowing the dissoluted silicate into the pores under weakly alkaline condition. Mesostructured silica solid spheres synthesized via the Stöber method using CTAB-TEOS composite formation in ammoniacal water-ethanol media have the characteristics of low degree silica condensation in the inner region when compared to outside. As a result, outmost layer of this silica is more compact than the inner. Based on this property, Teng et al. carried out the fabrication of ordered porous silica hollow sphere with radially oriented mesochannels, simply by a self-transformation approach followed by incubating this Stöber derived silica in pure water for certain time and at a particular temperature [58]. The rapidness of the hollow sphere formation, its size and shell thickness etc., is critically dependent on the water incubation time and temperature as well as ethanol/water volume ratios. A similar method was also employed by Lee et al., in order to fabricate hollow porous silica microcapsules having tunable diameter, inner volume, and radial mesochannels, suitable for encapsulating guest molecules [59].

Sometimes, quaternary ammonium silane N-trimethoxysilylpropyl-N,N,N-trimethyl ammoni- um chloride (TMAC) is used for the fabrication of positively charged silica hollow sphere in colloidal forms [60]. In this case, first solid silica nanoparticles synthesized via the reverse micro-emulsion method were used as template for hollow structure and it was then redispersed in water-ethanol mixture containing TMAC to modify its surface. Finally, the dissolution of core silica template in basic media starts simultaneously with the condensation of the TMAC on the surface, resulting in the formation of hollow silica sphere.

**Figure 6:**
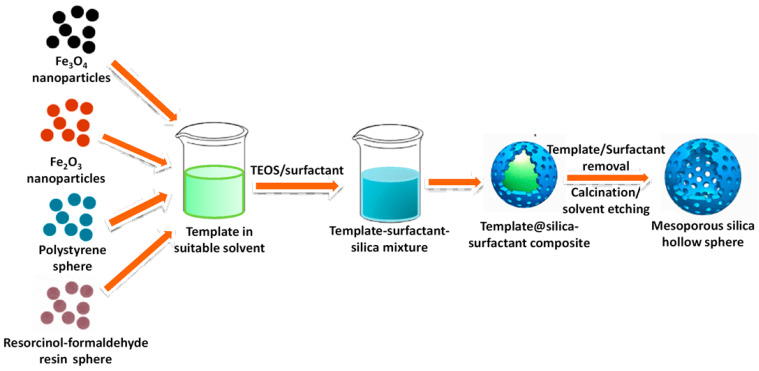
A comprehensive outline of hollow mesoporous silica (HMS) synthesis using various templates and CTAB surfactant.

Khanal et al. reported the use of polymeric surfactants micelle with core-shell-corona (ABC type) structure to synthesize hollow silica nanosphere [61]. Here, poly(styrene-*b*-2-vinyl pyridine-*b*-ethylene oxide) (PS-PVP-PEO) surfactant forms core-shell-corona type micelles in water-DMF media under weakly acidic condition, then, upon the addition of TMOS, the mixture was stirred and aged for hydrolysis of silica precursor. Finally, the removal of polymeric template from silica/PS-PVP-PEO composites by calcination produces hollow silica sphere. Unique structure of PS-PVP-PEO with hydrophobic PS core, ionic hydrophilic PVP shell, and hydrophilic PEO corona helps to form the desired morphology at optimized pH~4 [61]. By changing the size of the PS block chain or varying the silica precursor amount, the shell thickness, void volume of the hollow silica particles can be controlled precisely. A latex template can be utilized as core template for hollow silica synthesis [62]. When an ammoniacal solution of CTAB surfactant containing TEOS is added in a polystyrene-methyl acrylic acid (PMA) latex sphere aqueous suspension, it results in the formation of CTAB-silica composite over solid core. The removal of template generates mesoporous hollow silica nanoparticles [62]. The PS template has also been used by Nandiyanto et al., to fabricate mesopore-free monodispersed silica hollow sphere with smooth surface [63]. Blas et al., has also reported hollow silica synthesized while using polystyrene latex as core template and CTAB as contemplate to form mesoporous shell along with hydrolysed silica precursor [64]. Using an additive-free synthesis procedure, silica coating was made on the polystyrene template in presence of lysine catalyst, finally removal of template generates silica hollow sphere. The diameter of the hollow sphere and its morphology vary significantly with the size of PS template as well as with the TEOS/PS ratios. Pluronic F108 acts as single-micelle template for the synthesis of hollow porous silica nanosphere with very low silica precursor/surfactant ratio in the presence of a suitable organic swelling agent [65]. By adjusting the synthesis and hydrothermal temperature, silica precursor amount, the morphology, inner spherical void size, porous nature, etc., can be changed. Pluronic P123 can also be used for synthesis of hollow silica nanoparticles based on a modified SBA-15 synthesis method while using methyltrimethoxysilane as silica source instead of TEOS [66]. Rahman et al., have utilized various materials, say Fe_2_O_3_, Fe_3_O_4_, polystyrene, and resorcinol formaldehyde resin as core templates to synthesize mesoporous hollow sphere using TEOS in presence of CTAB or C_16_TMS surfactants [51]. Figure 6 illustrates a comprehensive outline of HMS synthesis using the above-mentioned method.

Different organofunctionalized silica precursors play a crucial role in the formation of silica hollow sphere [67]. Lin et al., studied and monitored the formation mechanism of silica hollow nanosphere in water-in-oil reverse microemulsion by in situ small angle X-ray scattering (SAXS) technique [68]. The aid of a polymeric surfactant Igepal CA-520, ammonia, and water in a continuous oil phase (alkanes), condensation and nucleation of ammonium catalysed silica oligomers from tetraethylorthosilicate (TEOS), and organosilane aminopropyltrimethoxysilane (APTS) collectively help to generate uniform silica hollow spheres. A detail discussion was presented also on the mechanism of formation of hollow sphere in microemulsion, its growth mechanism, effect of alkanes, surfactants, etc., to control the size and distribution of hollow silica nanoparticles. Another tunable hollow silica sphere of around 130 nm diameter was synthesized by a unique pathway [69]; first, the core was synthesized using TEOS and ammonia in water-ethanol media, then the core was coated with a disulphide functionalized mesoporous silica shell using TEOS, bis[3 -(triethoxysilyl)propyl] disulphide (BTDS organosilane), CTAB surfactant, triethylamine, and water-ethanol solvent. Subsequently, hollow silica nanosphere was obtained by the mild etching of this prepared silica particles with sodium carbonate aqueous solution [69]. Zhang et al., described the synthesis of magnetic Fe_3_O_4_ nanocrystal loaded hollow periodic mesoporous organosilica (PMO) while using CTAB and fluorocarbon (FC4) surfactants mediated cotemplating pathway [70]. Initially, Fe_3_O_4_ nanocrystals were fabricated in non-aqueous media, and then, by dispersing in aqueous CTAB solution, aqueous-phase dispersed Fe_3_O_4_ nanoparticles are generated. Finally, the co-condensation of two organosilanes, BTME and 3-aminopropyl triethoxy- silane (APTES), onto aqueous-phase dispersed Fe_3_O_4_ nanoparticles taken in CTAB-FC4 containing alkaline media develops magnetic nanoparticle loaded hollow PMO sphere. The morphology, size of the hollow sphere, shell thickness, pore structure, etc. can be altered precisely by adjusting the amount of FC4, organosilanes or Fe_3_O_4_ nanoparticles. Another approach for synthesizing magnetic Fe_3_O_4_ containing porous silica hollow spheres was taken by Wang et al. [71]. First, they prepared a yolk-shell superparamagnetic template composite via a one-pot synthesis process having miniemulsion polymerization and sol–gel reaction, and then coated it successfully with TEOS/C_16_TMS (hexadecyl trimethoxysilane) hybrid.

**Figure 7:**
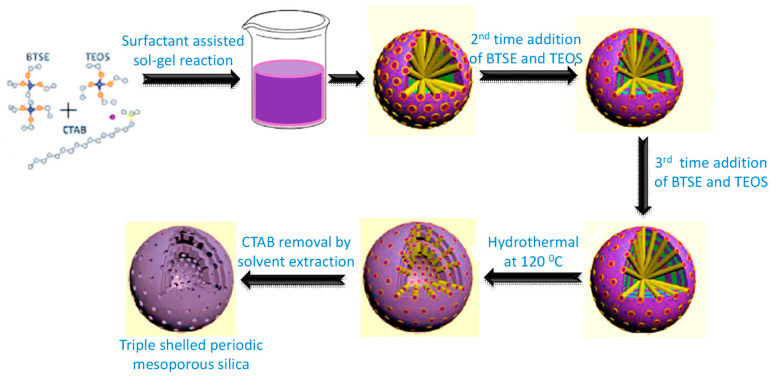
A pictorial representation of triple shelled periodic mesoporous silicas (PMO) formation using bis(triethoxysilyl)ethane (BTEE) as organosilane.

Hollow periodic mesoporous organosilica (PMO) spheres having organic functional groups into the inorganic shell with large cavities inside are a promising candidate for varieties of applications, like drug delivery, adsorption, and catalysis [51], etc. In 2015, Teng et al., proposed a simple strategy to prepare well-dispersed multi-shelled hollow PMO spheres via a “multi-interface transformation” approach by the stepwise addition of a mixture of TEOS and different high cross-linking organosilica precursors, like bis(triethoxysilyl)ethane (BTEE), 1,4-bis(triethoxysily)- propane tetrasulfide (TESPTS), or 1,4-bis(triethoxysily)benzene (BTEB) into the reaction media containing water-ethanol-ammonia-CTAB [72]. The number of shells formed depends on the addition times of silica precursors. A simple pictorial representation for the formation of multishelled PMO using BTSE is shown in Figure 7. In 2017, Yang‘s group has also successfully prepared double-shelled mesoporous organosilica hollow spheres having dendritic pore structure using CTAB and sodium salicylate as template, bis(triethoxysilyl)ethane and TEOS as precursors, followed by solvent extraction [73]. Recently, a template-free synthesis for nicely-designed multishelled hollow silica sphere, while using tetramethylammonium hydroxide (TMAH) as a basic catalyst, has been reported by Han and co-workers [74]. The periodical deposition of highly condensed silica layer on loosely condensed sacrificial silica layer makes a perfect volume ratio of 3:2 due to multi-injection of TMAH. By monitoring the amount of TMAH and batch number, the shell number, thickness and spacing between neighboring shell, etc., can be rationally controlled, which results in the formation of a perfect multishelled hollow sphere [74].

Zou et al., adopted a novel technique for synthesis of hollow PMO nanospheres based on organosilane-directed growth-induced etching mechanism. In a one-step route, growth of PMO shell occurs simultaneously with the etching and dissolution of its silica core template resulting hollow sphere morphology [75]. The diameter, shell thickness of the hollow nanospheres, etc., are highly dependent on the amount of organosilane added as well as on solid silica nanosphere size. Biodegradable disulphide bond (-S-S-) can be incorporated in hollow PMO shell by coating the solid silica sphere core template with a mixture of TEOS and BTDS, then by removing the core silica using soft etching process [76]. Other monodispersed biodegradable disulphide bridged hollow PMO nanoparticles with average diameter of ~90 nm have been synthesized by Rahmani et al., while using precursors bis(triethoxysilylpropyl)-methylamine (BMSPMA) and BTDS with CTAB surfactant in an alkaline oil-water microemulsion method [77]. Different kinds of organic groups can be incorporated into the hollow PMO shell using different bissilylated precursors with variable bridged organic moieties under the alkaline hydrolysis condition. PMO hollow sphere with highly ordered tunable hexagonal mesostructured wall has been synthesized by Djojoputro et al., by a new vesicle and a liquid crystal “dual templating” approach while using FC4 and CTAB as costructure directing agents and BTME as the hybrid silica precursor [78]. FC4 and organosilica self-assemble through a vesicle templating (VT) process to form the core; while, organosilica and CTAB composite micelles produce surrounding coating by a liquid crystal templating (LCT) process that determines the shell wall structure. Similar “dual templating” technique is employed by Qiao et al., to prepare PMO hollow spheres functionalized with different functional groups like –SH, –NH_2_, –CN, –C=C, –benzene, etc., while using the respective silica precursors along with BTME [79]. Highly elastic and flexible polyethylene can be incorporated in the shell structure of hollow silica nanospheres by using hyper-branched polyvinylpolytrimethoxysilane via soap-free oil in water (O/W) emulsion system [80]. High emulsifying capacity of this organosilica precursor helps to form hybrid polystyrene core−organosilica shell nanoparticles in the presence of polymerized styrene template and removal of the core via solvent etching yields the flexible polyethylene loaded hollow organosilica.

Very recently, an attempt to make PMO hollow nanospheres while using a block copolymer template has also been reported. A sol-gel method with a new pentablock copolymer template without additive pore expander was employed by Cho et al., to prepare hollow mesoporous organosilica nanospheres with a large pore cavity. Under acidic aqueous conditions, 1,2-bis(triethoxysilyl)ethane and 1,4-bis(triethoxysilyl)benzene were used as organosilica precursors and several kinds of PLGA-*b*-PEO-*b*-PPO-*b*-PEO-*b*-PLGA pentablock copolymers were used as structure-directing organic templates. Water-soluble pentablock copolymers were synthesized in the laboratory by attaching a more hydrophobic PLGA chain to both ends of Pluronic triblock copolymers (i.e., Pluronic F68 (EO_75_PO_30_EO_75_) and F108 (EO_141_PO_54_EO_141_)). A template was dissolved in acidic aqueous conditions and each organosilica precursor was mixed by stirring at 40 °C for 1 d, followed by aging at 100 °C, in order to obtain hollow sphere. The final mesoporous solid product was obtained after washing with HCl/EtOH and filtering with vacuuming. The cavity diameter of hollow nanospheres was varied mainly both by the acidic strength and the hydrophobic segment of the template. In the sol-gel reaction of organosilica materials with a template with a large fraction of hydrophobic segments and strong acidic conditions, the hydrophobic end of the pentablock copolymers was folded into a core part to form a single micelle structure, resulting in a hollow nanosphere with a large inner diameter up to 20 nm without a swelling agents. On the other hand, under the conditions of using a polymer with a small fraction of the hydrophobic chain or a weak acid, the terminal hydrophobic parts of the pentablock copolymer exist in an extended form, creating which inter-connected micelles, resulting in small pore network structure [81].

### 2.3. Core-Shell Silica Nanoparticles

**Figure 8:**
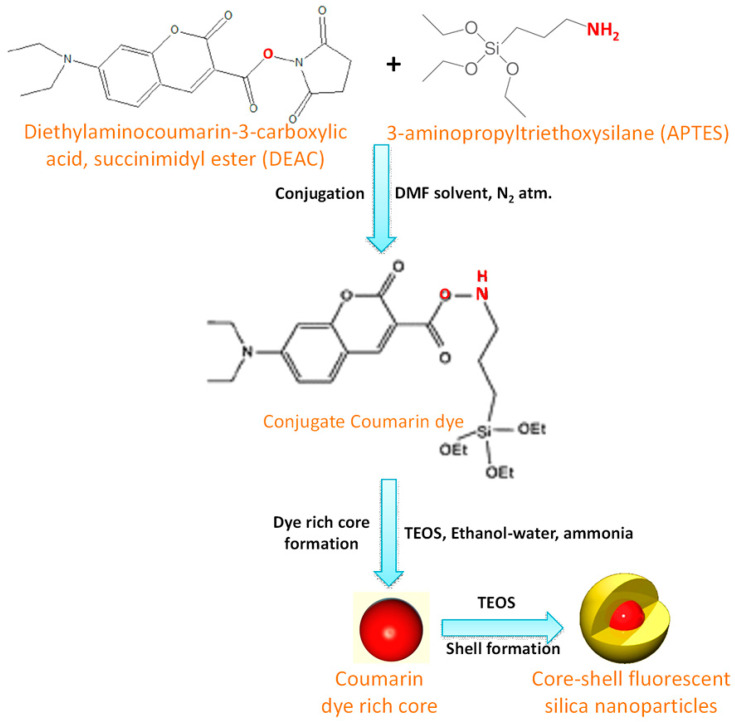
Formation of core-shell fluorescent silica nanoparticles using Coumarin dye rich core.

The core-shell structured material is a composite containing an inner core coated with one or more layers (called shell) of other materials [1]. For core-shell silica nanoparticles, either one of the composites or both of the materials of the composite is silica based structure [82]. Recently, core-shell silica nanoparticles have found enormous applications in sensing, bioimaging, energy application, catalysis, and so on [83]. In 2005, synthesis of highly fluorescent and monodispersed core-shell silica nanoparticles of 20–30 nm size has been reported by Ow and co-workers [84]. A dye-rich core was prepared using tetramethylrhodamine isothiocyanate dye and the silica shell was fabricated around it based on modified Stöber method. Herz et al. reported another fluorescent core-shell silica nanoparticle of 15–20 nm diameter, who adopted a three-step methodology for incorporating blue-emitting coumarin dye, 7-diethylaminocoumarin-3-carboxylic acid succinimidyl ester (DEAC) into the core of this nanostructure [85]. The dye ester was conjugated with APTES, then, following the Stöber method, TEOS was condensed with this conjugated molecule in ammoniacal water-ethanol media to form coumarin dye-rich core. Finally, pure TEOS was added over this core stepwise to form silica shell to protect the core within it. Figure 8 shows a schematic diagram for this core-shell particle. Synthesis of bifunctional colloidal mesoporous silica with core-shell structure can be accomplished by selective functionalization of outer shell or inner core of the particle with two different organo trialkoxysilane [86]. A unique delayed co-condensation strategy is applied here in order to control the condensation of silica precursors at different time of particle growth, leading to the formation of core-shell silica with different functional groups at the inner core and on the outer shell [85].

The above-mentioned synthesis methods for core-shell silica nanoparticles have mostly used cationic quaternary ammonium surfactants and basic medium. But Allouche et al., have reported a simple two-step sol-gel process for synthesis of core-shell silica while using non-ionic poly(ethylene oxide) type template like F127, Brij 97, Triton X-100 etc. [87]. Here, a porous silica shell is developed in weakly acidic media on solid silica core nanoparticles that can be prepared using modified Stöber method. The worm-hole pore structure and pore size of the silica shell can be tuned by changing the precursors concentration, types of surfactants, etc. Based on a combination of the Stöber method, Giesche growth process, and Kaiser approach, submicrometer-size monodispersed core-shell silica nanospheres has been developed by Büchel and co-workers [88]. On non-porous solid silica core, a porous thin shell is formed by adding a mixture TEOS silica precursor and n-octadecyl trimethoxy- silane pore generating agent called porogen. Finally, porosity is achieved by removing porogen by calcination, whereas inner core silica remains non-porous. The shell thickness is significantly dependent on the porogen concentration or TEOS amount.

### 2.4. Others Morphologies of Silica Nanoparticles

Beside the above-mentioned spherical, hollow, or core-shell nanostructures, some more interesting morphology-oriented synthesis of silica particles are also reported in literature. For e.g., Suteewong et al., have reported new mesoporous silica nanoparticles with branched shaped morphology synthesized using CTAB in presence of EtOAc and silica precursor [89]. The branches of hexagonally packed cylindrical pores can be grown in controlled way on silica with cage-like cubic porous morphology by monitoring the amount of EtOAc. In some cases, contemplate EtOAc can act as swelling agent to help form silica nanospheres of various morphologies. Occasionally, modified Stöber method utilizing n-hexane can generate ordered mesoporous silica with morphologies, like nanocubes, truncated nanocubes, microspheres, and twisted nanorods [90]. Simply, in the presence of CTAB template and TEOS silica precursor, and by varying the amount of pore expanding agent n-hexane, catalyst ammonia, these desired morphologies can be achieved. Even pure SBA-15 can be synthesized with numerous morphologies like fibre, rope-, doughnut-, sphere-, gyroid-, and discoid-like, etc., by varying cosurfactant, cosolvent, or silica precursors, etc. [91].

A beautiful 3D-dendritic mesoporous silica nanosphere with three generational center-radial dendritic channels, tunable pore size of 2.8–13 nm and thickness of 5–180 nm has been synthesized applying the oil–water biphasic stratification approach [92]. The surfactant C_16_TMAC, base TEA in aqueous media and TEOS in three organic phases say 1-octadecene, decahydronathalene, cyclohexane leads to the formation of three generational 3D dendritic silica. The variable size of pores clearly suggests the imperative role of organic molecules in the swelling behavior of this silica sphere [92].

**Figure 9:**
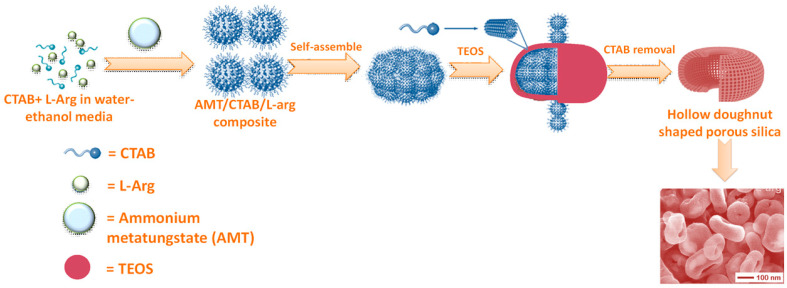
A simple scheme for synthesis of doughnut shaped mesoporous silica hollow sphere.

Fu et al., reported a simple strategy for the synthesis of hollow silica nanorods by using nanocrystalline cellulose as core template and forming a shell of CTAB-silica composite from an ethanol-water-NH_3_ media [93]. The removal of cellulose template by calcination results hollow nanorods. Unprecedented hollow doughnut shape morphology of mesoporous silica is observed when TEOS is added in water-ethanol mixture of CTAB and ammonium metatungstate hydrate (AMT) structure-directing agents containing L-arginine (L-Arg) amino acid as catalyst at room temperature [94]. Vo et al. investigated the role of AMT, amino acid, temperature, and solvent on this typical morphology. A simple synthetic route for this hollow doughnut shaped porous silica has been shown in Figure 9. A unique tumbler-like morphology of uniform magnetic/luminescent mesoporous silica nanoparticles has been synthesized by silica deposition on Fe_3_O_4_@SiO_2_ core-shell nanoparticles by Lin and co-workers [95].

Wrinkled silica mesoporous spherical nanoparticles can be generated in Winsor III type water–surfactant-oil bicontinuous microemulsion phase, containing CPB surfactant, TEOS precursor in water-cyclohexane-isopropanol [96]. The nanoparticle size, interwrinkle distance, etc., can be precisely controlled by changing the cosolvent type, volume ratio of cyclohexane/water.

Uniform spherical monodispersed organo bridged functionalized polysilsesquioxane nanoparticles can be developed via an inverse oil/water emulsion system [96]. Huh et al., studied the procedure and mechanism involved for different organofunctionalized mesoporous silica synthesis by the co-condensation of TEOS-organosilanes in NaOH alkaline media to achieve multiple morphologies like spheres, tubes, and rods, etc., depending on the types of organosilanes [97]. Ovoid shaped mesoporous silica nanoparticles can be obtained by room temperature condensation of dye-doped aminofunctionalized silica with cyano-bridged coordination polymer in methanolic media [98].

A novel discoid shape dye encapsulated ultrabright silica fluorescent nanoparticles have been synthesized by Palantavida et al., in acidic CTAB-dye aqueous media in the presence of sodium silicate as silica precursor and ethyl triethoxysilane as secondary precursor [99]. Similar type of work has been reported by Volkov et al., Presence of secondary silica precursor is important here in order to prevent the leakage of dye from the silica framework [100]. Another interesting report has been made by Chang and co-workers, who presented the synthesis of multishelled hollow mesoporous silica microspheres with single-shell, double-shell, and yolk−shell morphology in a CTAB-TEOS mediated water-ethanol-ammonia media [101]. These typical morphologies of silica microspheres can be simply obtained by controlling the amount of single reagent formaldehyde, which monitors the hydrolysis rate of silica precursor as well as the pore size of the template. Morphology-like dodecagonal tiling has been observed in quasicrystalline mesoporous silica nanoparticles synthesized using aqueous CTAB, ethyl acetate, ammonia, trimethyl benzene in presence of TEOS and organosilane 3-aminopropyltrimethoxysilane [102].

In addition, uniform shaped yolk-shell morphology of silica can be achieved, while Ni metal is loaded into silica framework using a CTAB-ammonia system in water-ethanol mixture to prepare Ni-doped silica nanosphere [103]. Using similar methodology and with higher amount of Ni species, NiO doped silica with flower-like monodispersed spherical particles have been obtained [104]. Additionally, the morphology is highly dependent on the Ni content in the material, because rod-like geometry predominates when Ni percentage increases in the silica structure.

The characterizations of the morphology-controlled mesoporous silica nanoparticles can be carried out using different analytical techniques [1,11]. Powder X-ray diffraction is used for mesostructure analysis, crystallinity of the materials, whereas information about nanostructures is obtained from transmission electron microscopy (TEM) and high-resolution transmission electron microscopy (HRTEM) is used for the determination of complete nanostructures, pore size. Particle shape or morphology study can be done while using FESEM recording. Quantitative analysis of the sample can be done by various analytical methods, like CHN analysis, energy dispersion spectroscopy (EDS)—elemental mapping, atomic absorption spectra (AAS), and atomic emission spectra (AES), etc. In addition, bonding connectivity between different atoms, oxidation state of metals, etc. have been analyzed by other techniques, like FT-IR (infrared), UV-visible, solid state ^29^Si CP MAS NMR, and X-ray photoelectron (XPS) spectral data.

## 3. Applications of Morphology-Controlled Mesoporous Silica Nanoparticles

Morphology-oriented silica nanoparticles have a wide range of applications in various fields, like catalysis [2], sensing [103], bioimaging [5], energy storage [4], etc., depending on the particle size, shape, pore size, and surface area of these porous nanomaterials. The different role of silica nanoparticles in numerous applied fields will be discussed in this section.

### 3.1. Catalysis

Because of the small size as well as large surface area, porous silica nanoparticles are always desirable as good support for heterogeneous catalysis [2]. Although silica solid spherical nanoparticles can be synthesized in numerous types of morphologies, but the application of pure silica spheres in heterogeneous catalytic transformation is quite restricted. When properly functionalized with any organic moiety or any transition metals, the material becomes proficient nanoreactor for catalytic and chemical transformations [2]. To date, considerable effort has been made by worldwide scientists for this research topic.

**Figure 10:**
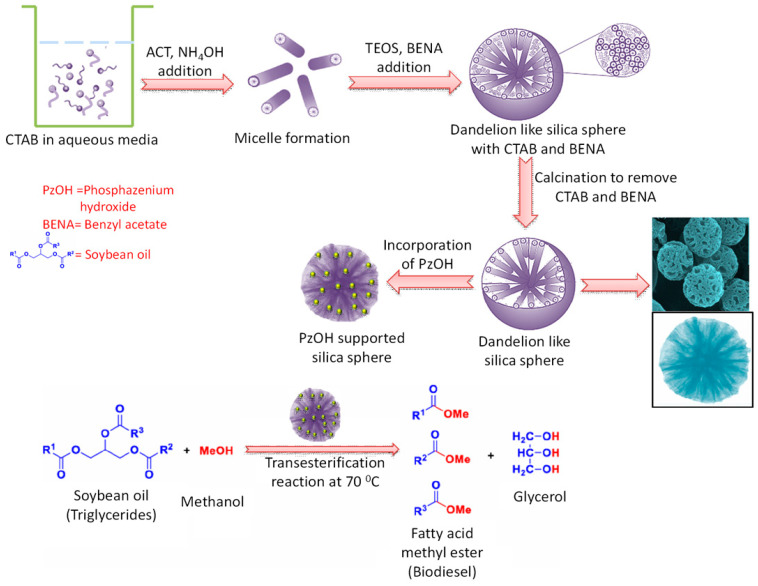
Synthesis of dandelion type silica sphere, PzOH incorporation, and catalytic transesterification reaction over it.

Mesoporous silica nanospheres with a wide range of particle size from 15–200 nm, synthesized using CTAB-basic amino acid has been incorporated with Ti metal in order to investigate the oxidizing property of the material [25]. On the other hand, Ti loaded ceria-silica spherical nanoparticles have exhibited an excellent role in the visible light induced photocatalytic oxidation and detoxification of organic dyes under mild conditions [105]. Liquid phase epoxidation reactions of small and large sized alkenes, like cyclohexene, *cis*-stilbene, and caryophyllene, have been carried out with this Ti-loaded silica catalyst under suitable conditions [25]. It was observed that small sized catalyst (25 nm) is more efficient for epoxidation of bulky alkene *cis*-stilbene and caryophyllene than the large sized one (70 nm). Uniform sized silica nanospheres with dendritic pore channels have proved to be very effective heterogeneous support for the reduction of 4-nitrophnol when the silica is loaded with gold nanoparticles [46]. It is suggested that well diffusion of reactants through dendritic channels of silica and uniform dispersion of Au active sites throughout the silica nanoparticles lead to high conversion percentage of 4-nitrophnol to 4-amino phenol. Mn loaded ceria-silica composite nanospheres that were synthesized in CTAB-ammonia- water-ethanol media have shown good performance for biofuel production leading to the transesterification of different esters under solventless conditions [106]. Uniform pores of this catalysts help in well incorporation of Mn species into the silica support resulting a significant yield in the presence of both long and short chain alcohols. The fibrous morphology of monodispersed dendritic silica nanospheres is extremely effective as heterogeneous support for catalysis, as the microstructures in this silica help in improving the accessibility of the active sites by the reactants, resulting in a high yield of products [29]. In this connection, Polshettiwar’s group has reported different transition metals, like Pd, Ru, etc., functionalized fibrous silica nanosphere for multiple organic reactions, such as Suzuki coupling reactions, hydrogenolysis of alkane, etc. [107]. Du et al., have also reported noble metal (Au, Pt, Pd) loaded amino-functionalized fibrous silica nanoparticles as a superior catalyst for reduction of 2-nitroaniline [108]. The open and flexible fibrous morphology has an excellent effect on catalytic property. This has been proved when nitridated silica fibrous nanoparticles show better activity as base catalyst towards for Knoevenagel condensation and transesterification reactions than the conventional SBA-15 or MCM-41 [109]. On the other hand, when phosphazenium hydroxide (PzOH) was supported on dandelion-type silica spheres synthesized using CTAB-ammonia-acetone media and benzyl acetate as pore modifier, the resulting materials exhibited brilliant response towards the transesterification of soybean oil in presence of excess methanol [110]. In Figure 10, the illustration of synthesis strategy and catalytic reaction over this PzOH-supported catalyst is shown schematically.

The unique structure of a hollow silica sphere having ordered mesoporous shell and large empty space in core can provide better platform for catalysis [14]. The confined space in the interior helps to load a large amount of active sites, whereas the porous shell facilitates well diffusion of reactants for easy access of the active sites. There are many reports and reviews in literature regarding catalytic applications of functionalized hollow silica nanospheres [14,52]. In 2004, Jin and co-workers prepared a high surface area Ni-silica composite hollow sphere with highly tunable shell thickness and used the material for hydrogenation of acetone [111]. When CuO is loaded in hollow silica sphere, the material became a suitable heterogeneous catalyst for the synthesis of 1,2,3-triazole compound via the click reaction in green solvent water [112]. Yang et al., have suggested an effective route for synthesis of large surface area hollow mesoporous silica (HMS) sphere in emulsion method using dodecylamine and TEOS emulsion droplets. The hollow sphere was loaded with Pd metal that exhibited a good catalytic activity towards phenol hydrogenation in mild conditions [113]. Pd loaded HMS is also an efficient and reusable catalyst for Suzuki reaction to produce high yield of diaryl derivatives [114]. Sometimes, a separate organic compound is used to capture the metal species. Kuwahara et al., have reported such type of material consisting Pd nanos and aminopolymers, poly(ethyleneimine) confined hollow silica spheres, which can act as an efficient reusable catalyst for semihydrogenation of alkyne-like phenylacetylene to produce *cis*-stilbene with more than 90% yield [115]. The strong poisoning effect of linear amine polymer helps in the high selectivity of the *cis* product. Noble metal loaded HMSs can show a wide range of catalytic performances for different chemical transformations. Ag@HMS nanoparticles have shown an imperative role of the unique hollow structure for catalytic reduction of 4-nitrophenol [115]. It was observed that catalytic activity increases with the increasing mesoporosity of silica shell favoring smooth transport of the reactants to access the encapsulated Ag nanoparticles. Ag functionalized doughnut shaped silica nanoparticles also can be used for such catalytic reduction process [116]. Wu et al., have reported the synthesis of Au@hollow silica monodispersed nanospheres using water-in-oil microemulsion method and the resultant nanocatalyst have been used for reduction of 4-nitrophenol showing good resistance towards other poison molecules like thiol compounds [117]. Additionally, Tang and co-workers have shown Pt loaded HMS sphere can be a highly active support for successful oxidation of volatile organic compounds [118].

**Figure 11:**
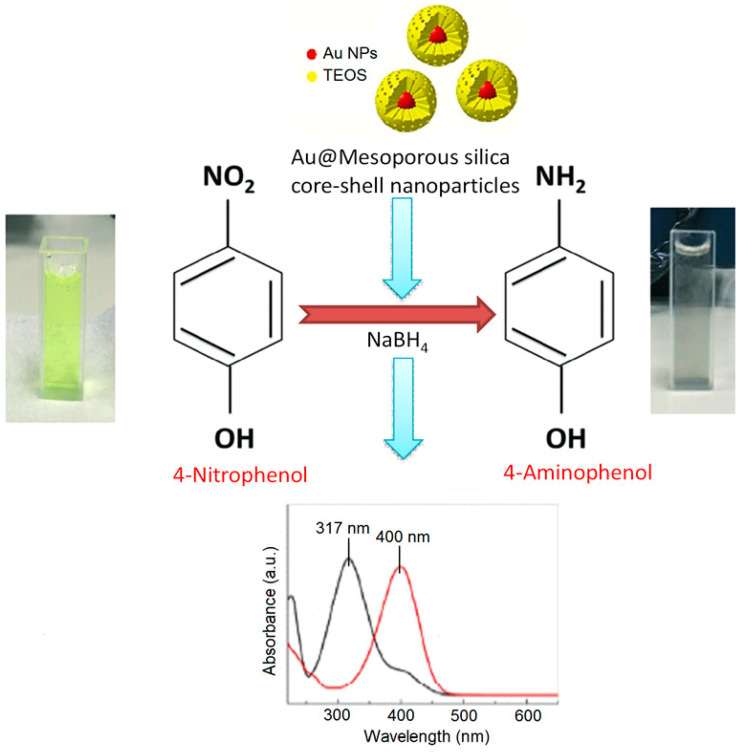
Catalytic reduction of 4-nitrophenol on Au@mesoporous silica core-shell nanoparticles.

A number of articles and reviews have been reported on the catalytic activity of organofunctionalized hollow mesoporous silica nanoparticles to date [1,14]. For instance, the hollow spherical shape and nanoscale size of a chiral amine modified SO_3_H-functionalized silica nanoparticles has been proved a determining factor for exclusive catalytic role of the material in asymmetric aldol condensation of cyclohexanone and 4-nitrobnzaldehyde with >90% yield under optimized conditions [119]. Verho et al., reported the Pd@amine-HMS catalyst for semi- hydrogenation of mono- and di-substituted alkyne to produce highly selective yield of alkenes [120]. The high catalytic performance and reusability of Au@amino-grafted hollow silica spheres were proved by the reduction reactions of 2-nitroaniline/NaBH_4_ and 4-nitrophenol/NaBH_4_ [121]. The sulfonation of a phenyl-functionalized hollow silica nanosphere can load ultra-high acid strength in the material, which, in turn, effectively shows catalytic performance in the condensation reaction of benzaldehyde with ethylene glycol in cyclohexane [122]. The high yield of the product significantly depends on the incorporated acid sites, and the catalyst is also actively reusable up to the 11th cycle of the catalytic reaction. Similarly, both acid and basic sites can be incorporated in core-shell structured silica materials, which can act as an active bifunctional catalyst for one-pot cascade reaction sequence to produce selective yield [123]. Using sodium aluminate in CTAB-Na_2_CO_3_ media solid silica sphere can be converted to hollow mesoporous aluminosilicate with perpendicular pore channels [124]. Instead of solid silica sphere use of Au@solid silica sphere can produce yolk-shell mesoporous silica that acts the role of a nanoreactor for 4-nitrophenol [124]. Additionally, the loading of Pd in this material facilitates its role for two-step reaction sequences to synthesize benzimidazole derivatives. The high permeability of hollow aluminosilicate shell allows for reactants to access the metal ions that are present in core making this HMS as a successful platform for catalytic reactions [124]. There are numerous reports on both heterogeneous catalytic and electrocatalytic applications of core-shell silica nanoparticles [83,125]. Main advantage of this morphology is tunable porous mesoporous shell which can protect the metal active sites present in core, allow the reactants to access the metal site and the products to diffuse out. PdNi alloy particles coated with mesoporous silica shell can form Pd–NiO@SiO_2_ core–shell mesoporous nanocatalyst which show superior catalytic role for the hydrogenation of p-chloronitrobenzene [126]. High selectivity and reusability of this bimetallic catalyst can be attributed to the interaction of Pd and NiO at their interphase in the core. Several core-shell silica nanoparticles loaded with precious metals like Pt, Ag, Au, etc., can show significant catalytic activity [127]. Joo et al., have suggested a Pt@silica core-shell nanocatalyst for high temperature CO oxidation reaction [128]. In other work, Pt loaded monodispersed core-shell nanosphere with dendritic large pores exhibited outstanding catalytic efficiency in hydrogenation reactions by converting nitrobenzene to aniline in just 2 h [129]. Similar catalytic reaction with 100% selective formation of aniline was also been investigated using Ag@silica core-shell mesoporous silica [130]. When tiny Ag nanoparticles are encapsulated within magnetic core-shell dendritic mesoporous nanosilica, the catalyst can be easily separated from the reaction mixture using strong magnet [131]. Sometimes, silica-encapsulated Au-Ag core-shell nanorod shaped catalysts can also be effective for catalytic reduction [132]. Similarly, Li and co-workers have loaded Pd into magnetic core-shell mesoporous nanosilica sphere which proved to be effective for Suzuki coupling reaction of aryl halides with phenylboronic acid and recoverable by applying an external magnetic field [133]. In another case, well-defined Au-loaded silica mesoporous core-shell has been synthesized using adjustable amount of HAuCl_4_ and tested for catalytic reduction of nitrophenol to aminophenol [134]. The reaction scheme for this reduction is shown in Figure 11.

Cu@SiO_2_ core-shell nanoparticles synthesized while using ultrafine Cu nano have been effectively utilized for hydrolytic dehydrogenation of ammonia borane (NH_3_BH_3_) and hydrazine borane (N_2_H_4_BH_3_) at room temperature [135]. It is observed that, under similar reaction conditions, nanospheres Cu@SiO_2_ exhibit superior catalytic performance when compared to commercial SiO_2_ supported Cu nanoparticles; SiO_2_ nanospheres supported Cu nanoparticles or free Cu nanoparticles. Pd encapsulated core-shell silica can also be used for water purification by reducing oxyanions type water pollutants [136].

Like core-shell architectures, porous yolk-shell structured silica nanoparticles have the wide advantage of protecting the metal species present as yolk with a silica coating and the facility of porous shell, which allows accessing the active sites by the reactants and diffusion of the products after the reaction [137]. Hence, a wide range of yolk-shell metal-silica nanoparticles as catalytic vehicles was reported in literature. Wei and co-workers have developed a new yolk-shell nanomaterial as promising catalytic system for biomass conversion consisting of Ru-supported carbon core encapsulated silica shell with radial mesochannels [138]. The yolk-shell particles show great efficiency for the one-pot hydrolysis-hydrogenation of dextrin to sorbitol in the presence of amyloglucosidase enzyme. Yao et al., have prepared a yolk-shell composite having a movable Fe*_x_*O*_y_* core and Pd nanoparticles embedded mesoporous SiO_2_ shell [139]. This stable Fe*_x_*O*_y_*/Pd@mSiO_2_ yolk-shell material has shown good reduction behavior for the catalytic reaction of nitrophenol in the presence of NaBH_4_. Similarly, single Au nanoparticles loaded yolk-shell silica microspheres can act as size-dependent heterogeneous catalyst for such reduction reactions [140]. Likewise, Ag/C entrapped mesoporous silica yolk-shell polymer-silica composites have also shown a promising role in nitrophenol reduction with >98% conversion rate [141]. In this connection, the reducing efficiency of another reusable and easy recoverable ellipsoidal shaped yolk–shell magnetic-silica structure with stable Au nanoparticles can be mentioned [142]. Mao’s group has developed mesoporous SiO_2_ yolk shell confined core-satellite Ag nanoparticles, which is proved to be highly efficient for catalytic reduction of Rhodamine B (RB) [143]. A high conversion efficiency and good selectivity is observed for selective oxidation of different alcohols over Pd encapsulated yolk-shell PMO nanomaterials prepared while using silica core and PMO shell having perpendicular mesochannels [144].

In addition, bimetallic catalyst like NiCe@m-SiO_2_ yolk-shell nanostructures has shown wonderful efficiency for CO_2_ reforming of methane [145]. Another catalyst is AuPt nanoalloy@SiO_2_ yolk-shell microsphere which shows good performance for styrene epoxidation as well as nitrophenol reduction [146]. Yolk-shell structured bifunctional catalyst synthesized by assembling amine functionalized silicate yolk as an inner core and chiral cinchonine-based squaramide loaded silicate as outer shell exhibits imperative activity towards the one-pot nitroaldol–Michael cascade reaction involving three-component coupling of nitromethane, aldehyde, and acetylacetone in order to produce various chiral diones with high yields and enantioselectivity [147]. Rajabzadeh et al., designed a new catalyst CuO@SiO_2_ multi-yolk@shell which shows good activity for CO_2_ fixation reactions say cycloaddition reaction of epoxides in presence of CO_2_ under solventless conditions [148]. Titanium silicate@mesoporous silica composites have been synthesized by Zou and co-workers, and the microstructured material shows high activity for hydroxylation of phenol in presence of H_2_O_2_ oxidant [149]. Wang et al. developed amine functionalized PMO@acid- functionalized silica yolk-shell nanoarchitecture, and the material acts as successful nanoreactor for one-pot deacetalization-Henry cascade reaction of benzaldehyde to produce high yield of benzylidenemalononitrile under mild conditions [150].

### 3.2. Biological Applications

**Figure 12:**
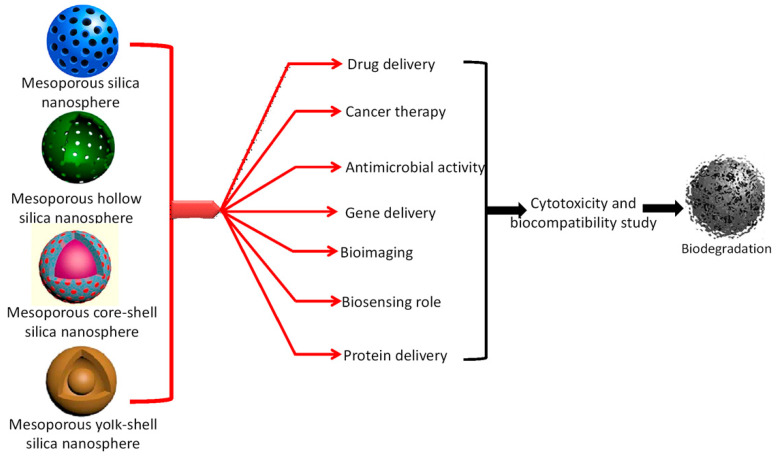
A schematic diagram for various biological applications of morphology oriented porous silica nanoparticles.

The interrelationship between nanomaterials and bioapplications is wide enough, because highly tunable pore structures, variable morphologies, and particle sizes of silica or functionalized silica nanoparticles facilitates versatile biological activities successfully carried out over this nanoplatform [14,151,152]. Applications, including biomolecule adsorption, drug delivery, cell imaging, enzyme immobilization, cancer therapy, etc., can be mentioned in this regard [151,152,153]. In literature, a huge number of reports has been found to be related to these biological and biomedical applications of porous silica nanoparticles as classified in Figure 12.

#### 3.2.1. Drug Delivery

The advantage of using porous silica nanoparticles as delivery vehicles for different drug molecules is the flexible porous structure that enables encapsulating different drug molecules to transport them to the target site by protecting from the enzymatic degradation. Drug molecules are simply adsorbed into the silica particles by soaking them in drug solution [151]. The interaction is through electrostatic and hydrogen bonding. The drug loading capacity is highly related to the surface area, pore size, and morphologies. Therapeutic capability also depends on proper functionalization of silica nanostructures that facilitates essential drug loading and controlled release at the target tissues reducing the adverse side effect of drugs in the body [152,153].

Amine-functionalized silica nanosphere is a promising vehicle for delivery of a representative hydrophobic anticancer drug, camptothecin (CPT), into human cell to induce apoptosis or death of cancerous cell [154]. In another work by Chen et al., Fe_3_O_4_ nano-capped mesoporous silica spheres has been used as site specific, leakage free, and superior nanocarrier for CPT drug delivery, as the drug release is controlled by external magnetic field that removes the Fe_3_O_4_ nano-caps by chemical bond breaking and leads to fast release of the drug at target tissues [155]. Depending upon the strength and duration of magnetic induction the dosage of anti-cancer drug can be applied in the particular cancer cell. Similarly, surface functionalization of magnetic Fe_3_O_4-_silica nanoparticles with different organic functionalities, like –COOH, –NH_2_, –Ph, –PO_3_^−^, etc., can increase the potential of silica nanoparticles for loading and controlled release of anticancer drugs, like doxorubicin hydrochloride (DOX) and paclitaxel (PTX) for tumour therapy [156]. The loading of proper sonosensitizing functional groups and metals onto mesoporous silica can make it a highly promising candidate for sonodynamic cancer therapy. In 2017, Huang et al., have reported paramagnetic metal say Mn entrapped protoporphyrin complex loaded onto mesoporous silica pores [157]. This Mn functionalized sonosensitizing material shows excellent performance for high magnetic resonance imaging enabling successful sonodynamic therapy to induce in-vitro cancer cell death [157]. Curcumin based colloidal fluorescent PMO nanoparticles that were synthesized by Datz’s group exhibit good biocompatibility and stability in simulated body fluid (SBF), showing significant potential for drug delivery applications [44]. Cheng’s group synthesized pH-sensitive trimethylammonium (TA) group encapsulated mesoporous silica nanoparticles that can load a high amount of oral anionic drugs and deliver it to colon. pH-sensitive hydrazone bond breaking of TA groups enables the complete and fast release of adsorbed oral drug in colon at pH 7–8 [158]. Sometimes, mesoporous silica nanoparticles (MSN) with surfactant CTAB can exhibit high efficiency as anticancer drug. Even when compared to common anti-cancer drug CPT-11 loaded surfactant free, CTAB@MSN shows more activity towards long-term anti-cancer efficacy [159].

The unique role of hollow mesoporous silica (HMS) and organosilica in drug delivery is noteworthy to mention here [151,160]. Typical hollow interior of silica particles helps to encapsulated large amount of drug molecules whereas tunable mesoporous outer shell ease the controlled release of drug molecules at the target site. Very recently, Poostforooshan and co-workers have fabricated aerosol-mediated hollow silica mesoporous nanosphere which after being functionalized with poly(allylamine hydrochloride) and alginate as biocompatible polymers, shows the high efficiency for loading of an antibiotic drug amoxicillin and its delivery at the desired site [54]. Because tumour cells are more acidic than the normal cells, by grafting with pH-sensitive chitosan, HMS exhibits successful incorporation of an anticancer drug pro-apoptotic NCL antagonist agent (N6L) and pH-responsive drug release at pancreatic cancer cell, inhibiting cell growth up to 60% [161]. Similarly, glutathione sensitive HMS nanoparticles have been used as an ideal biodegradable drug delivery vehicle by loading doxorubicin drug at the large void space of hollow sphere and releasing it in controlled way at cancerous cells [69]. HMSs synthesized with various core-templates have been used for the adsorption of anti-inflammatory drug ibuprofen and its sustained in-vitro release study in SBF solution [51]. Employing Au nanoparticles and CTAB as dual templates, hollow mesoporous silica with a highly tunable inner core and porous shell thickness were prepared by Li’s groups [162]. The unique and flexible structure of this material proved to be essential as a nanocarrier for the adsorption of doxorubicin drug and its controlled in-vitro release study.

**Figure 13:**
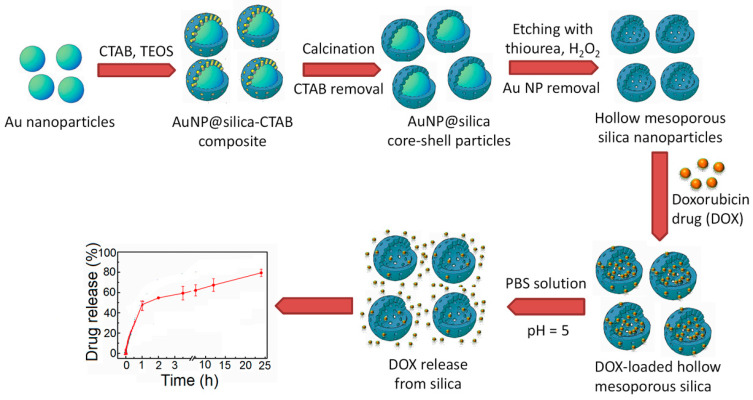
Loading of doxorubicin hydrochloride (DOX) drug onto hollow mesoporous silica and its release efficiency.

Figure 13 illustrates the synthesis scheme for this HMS and its *in-vitro* drug delivery activity. Multishelled HMS spheres because of more heterogeneous interfaces are expected to show a better performance than the single shell HMS. Cao’s group developed a luminescent double-shelled HMS by using CTAB and the material was very efficient in loading of pyraclostrobin pesticide and sustained release of the pesticide at definite location [163]. This property of silica nanoparticle can be applicable for plant protection strategy while using pesticides.

Core-shell silica materials are more desirable for biomedical applications because, these specific morphology leads to less toxicity in physiological system making a non-toxic coating of silica onto core surface, increase the chance of dispersibility by providing a hydrophilic coating to a hydrophobic core, also inert highly stable silica enhances the thermal and chemical stability of the core-shell nanoparticles [152,153]. This is clearly observed when silica xerogel is used as core stabilizer to prevent drug leakage in a silica-polymer composite which monitors controlled and prolonged release of vancomycin, an antibiotic drug release at in-vitro PBS solution [164].

Another interesting work by Shi et al., reports the construction of thermosensitive peptide-coated ZnO@Fe_3_O_4_-silica porous core-shell nanoparticles based on microwave irradiation [165]. Modified peptide in this material entraps the drug molecules onto the pore surface of silica at physiological temperature and releases them by opening the pores at elevated temperature. Role of ZnO@Fe_3_O_4_ is to absorb microwave radiation, which on controlled penetration into the cell tissue induces thermoresponsive drug release [165]. In similar way, Saint-Cricq and co-workers have synthesized a novel azo-functionalized thermodegradable poly(ethylene glycol) coated Fe_3_O_4_@SiO_2_ core-shell MSNs nanocarrier which can release sample drug molecules by breaking the azo bond when temperature is elevated within a biologically relevant narrow temperature range by applying an external magnetic field [166]. Amirthalingam et al., have synthesized a novel drug delivery vehicle silica-gold core nanoshell material by attaching NH_2_-functionalized silica with monodispersed gold nanoparticles [167]. The material was loaded with an antibiotic drug Gentamycin with a maximum amount of 87 μg/mg and the release profile has been studied on gradual release of drug molecules by breaking of Au- Gentamycin coordinate bond.

Yolk-shell silica nanostructures or silica ‘nano-rattles’ consisting of metal based nanoparticles core inside mesoporous silica shell are one of the most promising candidates for drug delivery system [168]. Out of several reports in literature, Fe_3_O_4_@PMAA composite by Zhao et al. [169], bifunctional magnetic silica nanostructure by Huang et al. [170], iron oxide@magnesium silicate nanosphere by Sun et al. [171], yolk-shell nanostructure reported by Dai et al. [172] are worthy to mention. Yolk-shell structured triple hybridized PMO upon loading of ethane-, thioether-, and benzene-bridged moieties can show multiple applications like bioimaging, biocompatibility, drug delivery etc. owing to the bonding with fluorescent dye, ability to load high amount of hydrophobic drugs as well as glutathione-sensitive drug release capacity [173].

Beside these, delivery systems for other essential biomolecules like genes, antigens, proteins using morphology-oriented silica nanoparticles are also reported by various scientists’ groups [57,151,152,153].

#### 3.2.2. Bioimaging

In order to study the biological process in living system or following the behavior of drug molecules and monitoring the progress of infected cells, optical imaging or bioimaging is an important topic [153]. In recent decades, a huge progress is observed on bioimaging study using morphology-controlled silica nanoparticles [174]. Liu and co-workers have successfully developed a RB grafted fluorescent mesoporous silica nanoparticles as an nontoxic bioimaging platform to selectively detect Cu^2+^ in solution as well as in living cell while other interfering agents like Hg^2+^ is present [32]. Cu^2+^ promotes the release of RB dye from silica by hydrolysis in absence of Hg^2+^. Ow’s and Herz’s group have fabricated a stable fluorophore grafted core-shell silica nanoparticles which can be used for labeling of different biomolecules in bioimaging applications [84,85]. Palantavida et al., have synthesized novel folic acid functionalized ultrabright mesoporous fluorescent silica nanoparticles, which can differentiate accurately between cancerous and precancerous cervical epithelial cells from normal healthy cell depending on the fluorescence property [175].

On the other hand, core-shell structured silica nanoparticles embedded with phosphorescent iridium(III) complexes at core display a sharp luminescence property to sense exogenous and endogenous hypoclorite, ClO^−^ in live cells [176]. Fan et al., have reported a core-shell rattle-structured nanotheranostics, which shows a breakthrough in cancer treatment by a synergetic role as chemo-/radio-theraping agents and probe for luminescent dual-mode imaging upon loading and delivering cisplatin anti-cancer drug at tumour cell [177]. Bioimaging study was also performed using a fluorescence probe N, N-phenylenebis(salicylidebeimine)dicarboxylic acid grafted dendrimer type mesoporous silica nanoparticles with hierarchical pores [174]. When folic acid is conjugated to this compound, the material can perform as perfect fluorescent probe in potential bio-imaging study.

#### 3.2.3. Biocompatibility and Cytotoxicity

While using a nanomaterial in practical biological applications, the compatibility with living cell is an important point to be considered. A biocompatible substance should not produce any toxic effect or immunological response to the body. Mesoporous silica nanostructures have acceptable biocompatibility and low toxicity, which have been tested and reported several times in previous literature [151,152,153]. Hence, whenever a new material is used for clinical applications, cellular toxicity or cytotoxicity and biocompatibility should be thoroughly investigated. Some reports tell that mesoporous silica nanoparticles above 100 nm are less toxic than the sub-micron size (50 nm) particles which may cause necrotic cell death. Additionally, functionalized silica nanoparticles modified with amine or thiol functional groups have lower cytotoxicity than the unmodified MSNs [178]. If toxic surfactants, like CTAB, are not completely removed from MSNs before applying in living body, silica with residual surfactant present in its pore channels may cause much toxicity in the biological system [152]. A spherical nanocomposite double shell Fe_3_O_4_ cluster@nonporous SiO_2_@mesoporous SiO_2_ made of nonporous silica shelled magnetic Fe_3_O_4_ cluster as core and coated by a second mesoporous shell is tested for biocompatibility and cytotoxicity effect [179]. Because CTAB has been completely removed by high temperature the chance of toxicity from this material is reduced. Additionally, in-vitro viability test carried out with various concentrations of magnetic silica nanoparticles on DU-145 cell indicates less cytotoxicity effect and high biocompatibility [179]. Core-shell mesoporous silica nanoparticles composed of solid colloidal silica core and thin coating of porous silica shell offers less toxicity than the corresponding nonporous colloidal silica particles when interacting with human THP-1 macrophages [152]. Porous silica shell has greater number of proteins, which mask the surface silanol groups at silica-cell interface, thus reduce the toxicity level and inflammation. Consequently, mesoporous core-shell nanoparticles have improved biocompatibility that is suitable for any biomedical applications.

#### 3.2.4. Biodegradation

A lot of research works are going on about the biodegradation behavior of mesoporous silica based nanoparticles while applying them frequently as intravenous drug carrier in living cell [76,92,180]. A low biodegradation rate will increase the risk of silica accumulation in the body. Thus, the safe elimination of these nanomaterials with high degradation rate in simulated body fluid is quite desirable. Conventional mesoporous silica may take about more than one month time for complete clearance and, within this time, the nanoparticle accumulation may affect liver and spleen tissue [151,152,153]. A faster degradation rate can be achieved using colloidal silica nanoparticles of large pores, thin pore walls and low cross-linking degree. 3D-dendritic mesoporous silica nanospheres synthesized by Shen’s group have large pores and thin walls that facilitate the rapid simulated degradation of the particles in SBF solution within 24 h [92]. By incorporating physiologically active disulphide (-S-S-) bonds into a hollow mesoporous organic-inorganic hybrid silica nanocapsules, the degradation process can be improved. The degradation of porous silica nanoparticles depends on various factors, like its in-vitro concentration, its morphologies, particle size, pore size, etc. For any drug delivery experiments, a highly diluted aqueous solution must be taken with prior dissolution studies of the material. MSNs can be non-toxic when used up to 100 µg/mL concentration for in-vivo experiments. Möller et al., have done a literature survey to understand the influences of silica network connectivity, synthesis pH and co-condensation time, surface functionalization with organic groups, etc., on the dissolution and degradation of silica at low concentration [181]. Ratirotjanakul’s group has shown that the degradation of mesoporous silica can be accelerated by incorporating amino acids, like glycine, cysteine, and aspartic acid, into mesoporous silica framework [182]. They observed that nucleophilic side chains paly a decisive role for the amino acid functionalized silica degradation and aspartic acid having two carboxylic acid groups show highest degradability and lowest toxicity among all of the samples. Shi et al., have investigated the degradation behavior of three types of silica nanoparticles say core-shell composite, hollow sphere, and multi-layered for in-vivo and in-vitro bioanalyses [183]. It is predicted that intracellular fate of silica-based nanomaterials is highly dependent in the physicochemical considerations.

#### 3.2.5. Antimicrobial Activity

Bacterial infection in human body is very common and the most common organs for their entry are lungs, skins and intestine. *E. coli*, *V. Cholerae*, etc., are some well-known bacteria causing infectious disease in our body [54,77]. Antibiotics are used to kill the bacteria and remove them from our body. Morphology oriented silica nanoparticles are widely used as promising nanocarriers to deliver the antibiotic drug molecules to bacteria infected tissues. Silver nanoparticles have a pivotal role in increasing the antimicrobial activity of mesoporous silica nanoparticles [184]. Very recently, Rahmani’s group has reported an efficient hollow mesoporous organosilica nanoparticle, which, upon simultaneous loading with amoxicillin antibiotic and Ag^+^ ions, shows a significant antimicrobial role by inhibiting *E. coli* bacteria growth [77]. The effect of size and shapes of the nanoparticles have a keen effect on the antibacterial activity of Ag nanoparticles loaded and chitosan coated porous silica nanoparticles. The material was tested with both types of bacteria (gram-positive and gram-negative) and it was observed that *E. coli* bacterial growth can be inhibited by organosilica having a decent ratio of Ag and chitosan. Chen et al., suggested that use of core-shell ZnO@mesoporous silica nanoparticles along with nonporous silica as bimodal filler to prepare dental composites, improves mechanical and antimicrobial properties [185]. The presence of amine groups on silica enhances antibacterial efficiency in many folds. In 2015, Hao and co-workers have utilized NH_2_-functionalized hollow mesoporous silica for antibiotic and cancer drug loading that exhibit significant killing efficiency against mycobacteria (*M. smegmatis* strain mc^2^ 651) [186]. Amine functionalization, sufficient hollow void space to carry drug molecules, porous ~20 nm thick shell to release the drug molecules can be attributed to high anti-microbial activity of this material.

#### 3.2.6. Biosensing Study

**Figure 14:**
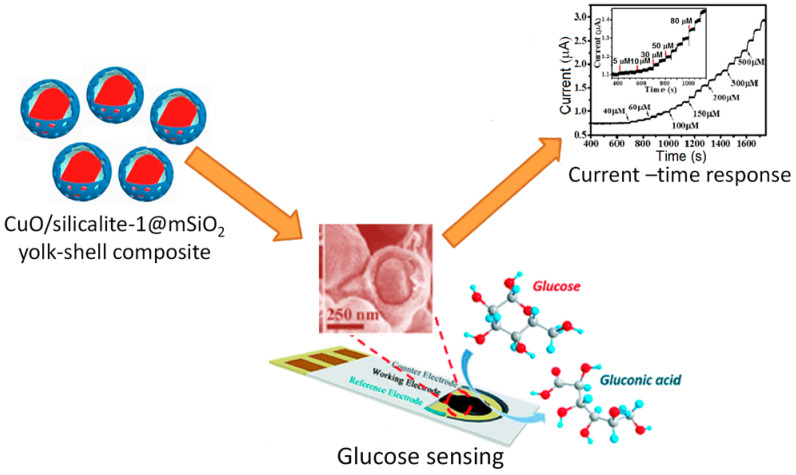
A schematic representation for glucose sensing activity of CuO/silicalite-1@mSiO_2_ yolk-shell composite.

Mesoporous silica nanoparticles, when properly anchored with suitable metal species, can show good potential as enzymatic or non-enzymatic biosensing property for different biomolecules, like glucose, hydrogen peroxide, etc., via electrocatalytic pathway [83]. Glucose detection is highly significant in biotechnology, food industry, as well as for diagnosis of blood sugar level in medical application. Ni metal shows the spontaneous tendency to response in electrocatalysis owing to the active redox couples Ni^2+^/Ni^3+^ [103]. Bimetallic Ni-Co alloy nanoparticles when loaded into the porous nanostructures of mesoporous silica, the resulting material has been proved as biosensing platform showing outstanding performance towards the non-enzymatic electrooxidation of glucose molecules [187]. With a high sensitivity of 536.62 μA mM^−1^ cm^−2^, the material shows good reproducibility, high selectivity, and long-term stability for glucose sensing in presence of other oxidisable interfering agents, like ascorbic acid, dopamine, and uric acid. Ni incorporation in yolk-shell nanostructure produces Ni-doped silica mesoporous nanoparticles, which exhibit excellent activity for enzyme-free glucose sensing in 0.1 M NaOH solution [103]. Cheng et al., prepared a novel yolk-shell nanostructured CuO/silicalite-1@mSiO_2_ composite by incorporating silicalite-1 supported CuO nanoparticles into mesoporous silica hollow sphere [188]. The material was highly selective for glucose sensing under non-enzymatic conditions with anti-interfering ability. The mesopores of this composite filled with glucose molecules can get proper access to Cu metal ions, which is essential to oxidise glucose to gluconic acid, whereas the large interfering and other biomacromolecules are inhibited to enter into the void and interact with active site. Due to this remarkable and interesting nanostructure, high electrocatalytic activity is observed with a wide range of linearly (5–500 µM), good sensitivity (5.5 μAmM^−1^cm^−2^), and very low detection limit (0.17 μM), which is required for practical glucose detection in human blood serum [188]. Figure 14 shows a schematic representation.

On the contrary, the detection of H_2_O_2_ is important to get information about its role in physiology for e.g., increased amount of H_2_O_2_ in heart may often cause chronic heart failure [189]. Dendritic mesoporous silica nanoparticles can be employed for electrochemical detection of H_2_O_2_ in living cell upon immobilization of horseradish peroxidase enzyme [189]. This morphology controlled porous silica nanoparticles have the advantage of large pore volume, highly accessible internal surface area which facilitates higher amount of enzyme loading and bioactivity compared to nonporous silica. Enzyme loaded dendritic silica nanoreactor when adhered on glassy carbon electrode shows extreme potential for selective and sensitive detection of H_2_O_2_ in pH = 7 phosphate buffer solution with a very low detection limit of 0.11 µM [190].

Another material, Ag loaded mesoporous silica nanoparticles, is also very much effective for electrochemical enzyme-free H_2_O_2_ detection selectively in the presence of other interfering agents, like glucose, ascorbic acid, and uric acid [190].

Phenol detection is important because most of the phenolic compounds are toxic and their accumulation causes serious damages to living cells and health of the body [191]. Magnetic Fe_3_O_4_ loaded nanocomposite biosensor has been developed using core Fe_3_O_4_ nanoparticle coated with mesoporous silica shell for phenol detection [192]. The resulting material is subsequently grafted with amine functionality, which, on condensation with an aldehyde, can incorporate tyrosinase enzyme by conjugation. This tyrosinase linked magnetic core-shell nanocomposite is very easily adhered on a magnetic electrode without any adhesive agents and catalyze electrochemical oxidation of phenol to produce quinone with high selectivity, sensitivity and very low detection limit [192]. In other case, flower-shaped yolk-shell silica nanospheres have been synthesized by Zheng et al., who utilized the material as enzymatic biosensor for electrochemical detection of catechol [193]. The unique radial mesochannel of the yolk-shell nanospheres, large surface area, cavities and thin mesoporous shells help to immobilize a higher amount of laccase enzyme and detect catechol with high selectivity and low detection limit of 1.6 μM in a wide linear range from 12.5–450 μM over catechol concentration [193].

Additionally, structurally controlled mesoporous silicas as an effective support for enzyme immobilization have also been utilized in many biocatalytic platforms [194,195]. For e.g., a keen investigation was made by Lee and his co-workers to use enzyme immobilized Fe_3_O_4_-silica porous nanospheres for the sequential conversion of cellulose to glucose to fracture [196]. Moreover, when functionalized with vinyl group, cubic mesoporous silica can act as a good support to increase the stability of cellulose enzyme, which is highly crucial for biocatalytic performances [197]. Even, many scientific groups throughout the world have been extensively engaged in the study to find out the effect of templates, solvents, and other factors on the immobilization of important enzymes on mesoporous silica nanoparticles [198].

### 3.3. Energy Storage Application

Because to its highly tunable uniform pores, thin pore walls, large surface area, and nano-sized particles, mesoporous silica nanoparticles are advantageous for various energy applications of this material, including supercapacitors, fuel cells, rechargeable batteries, and solar cell [4]. The mesopores of silica and the void space of hollow silicas facilitate the adsorption of large amount of guest molecules on the outer and inner surface of the materials, which is required for energy storage. Uniform mesochannels can help transport of ions, atoms, or molecules through the materials increasing the chance of accessibility of active sites, and the small mesopore channels minimize the transport pathway for electrons and ions, which is highly essential for solar cell, batteries, and water splitting reactions [4]. Mesoporous silica nanoparticles upon loading with semiconducting metals or functionalizing with organic groups etc., will exhibit good energy storage properties.

In supercapacitor devices (SCs), electrochemical energy storage is can be done by preparing the electrode materials with silica and other nanostructured materials. Mesoporous silica wrapped with GO and conducting polyaniline nanowires has been used as efficient hybrid electrode for supercapacitor [194]. When compared to the individual material like silica, polyaniline this composite shows high supercapacitive behavior (specific capacitance 412 F g^−1^), which can be attributed to the synergetic effect of mesoporous silica, GO and conducting polyaniline. The presence of graphene increases the structural stability, and the conducting and interconnection between all the particles [199].

**Figure 15:**
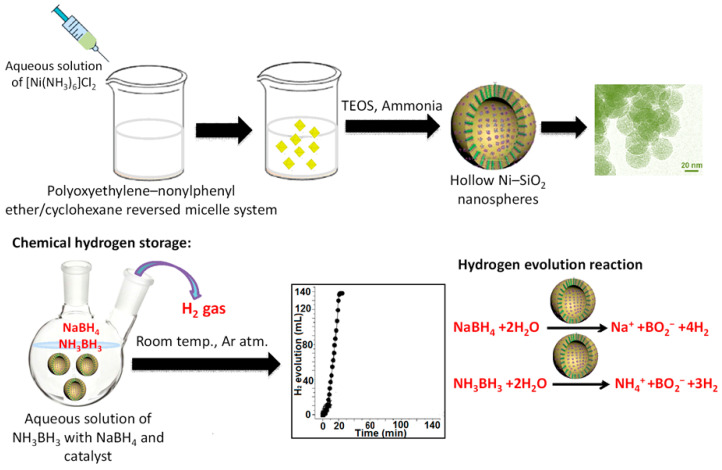
A schematic representation of catalytic hydrogen storage process over hollow Ni-SiO_2_ nanosphere.

With increasing demand for alternative and renewable energy sources in modern world, hydrogen storage and use of hydrogen fuel cell has become highly popular as non-toxic, convenient, and inexpensive [195]. Formic acid can be used as chemical hydrogen storage material for portable fuel cells as it contains about 4.4% (*w/w*) hydrogen. The decomposition of formic acid (HCOOH→H_2_ + CO_2_) is widely investigated now-a-days for hydrogen generation [200]. Yadav and co-workers have proposed a heterogeneous support consisting Pd loaded mesoporous silica nanosphere which shows high catalytic performance for formic acid decomposition in aqueous media under mild conditions. The interaction of Pd with silanol groups of the silica support is one of the crucial reasons for high catalytic activity of this material [200]. Similarly, hollow Ni-silica nanosphere synthesized by incorporating Ni clusters inside mesoporous silica, is able to catalyze efficiently the hydrolytic dehydrogenation of ammonia borane to generate hydrogen [201]. Thus, Ni-silica hollow nanosphere can act as perfect chemical hydrogen storage device and it shows higher activity than the conventional Ni supported silica catalyst. In Figure 15, a schematic representation of this catalytic hydrogen storage process is shown clearly.

Dye-sensitized solar cell (DSSC) is a well-known alternative energy source in present time. TiO_2_ as a semiconducting material has great possibility in dye-sensitized solar cell. But the efficiency of TiO_2_ in solar cell is highly dependent on the particle size and surface area of the material [202]. Hence, surface area of commercially available titania can be increased by making a composite using high surface area stable silica nanomaterial. In this connection, role of TiO_2_/SiO_2_ nanocomposite synthesized in a sol-gel process by Kumar and his coworkers can be mentioned. It is observed that TiO_2_/SiO_2_ nanocomposite has higher surface area than individual TiO_2_ nanoparticles and has shown high photocurrent enhancement in field-dependent photoconductivity. Thus, the material can be a potential candidate for an effective electrode for DSSC [202]. In another work, a plastic based dye-sensitized solar cell is constructed using quasi-solid-state gel-electrolyte of mesoporous silica nanoparticles, which has strong light scattering property in the visible region. Ionic conductivity and light-to electricity conversion efficiency of this silica is improved by allowing I^−^/I_3_^−^ diffusion through it [203].

Very recently, Wang et al., reported a novel method for preparing energy storage material using vertically aligned mesoporous silica thin films [204]. A functionalized transparent mesoporous silica thin film was prepared on indium tin oxide electrode surface. The high surface area of this silica film facilitates incorporation of redox active system say ferrocene into the silica framework. High charge transfer rate is possible through electron-hopping process and mass transport through the pores of silica. Thus, when combined with a graphene electrode, this functionalized silica thin film electrode is able to form a solid-state–battery–capacitor hybrid device [204]. The use of mesoporous silica for thermochemical energy storage, that is, storage of thermal energy by means of reversible reaction for future purpose by using salt hydrate is reported by Shkatulov et al. [205]. Because of the high energy density and storage duration salt hydrates, like LiCl·H_2_O, CaCl_2_·6H_2_O, SrBr_2_·6H_2_O, etc., can be used for domestic thermochemical energy storage purpose. Salt hydrates are encapsulated within hollow silica capsules, which facilitate easy mass transport from the capsule shell through its pores which is an essential criterion for constructing a thermochemical energy storage system. Investigation was carried out with different salt hydrates on the basis of cyclic stability and energy storage density, out of which Li salt has been proved to be promising candidate for energy storage at domestic level [205].

Some other applications of morphology controlled silica nanoparticles are also reported in literature. Due to high surface area and porosity, adsorption property for removing toxic dye molecules, like RB from aqueous solution, is very significant, which can be applied for water purification process [29,71]. Purification of microbes contaminated water can be done using Ag encapsulated hollow silica nanosphere [77]. Li and Ca doped ordered mesoporous silicas have been sued as effective adsorbent for H_2_ and CO_2_ storage at ambient conditions [206]. On the other hand, nitrogen rich core-shell magnetic silica nanoparticles can act as efficient adsorbent to remove Ag particles from water [207]. Mesoporous silica nanoparticles can also be used for corrosion protection upon change in pH of the environment [62].

## 4. Summary and Future Prospect

In a nutshell, this review enlightens the readers with the knowledge about the synthesis of various pure silica, organofunctionalized, and metal loaded silica nanoparticles of interesting morphologies ranging from nanosphere to hollow sphere, core-shell, yolk-shell, etc. The contributions of these morphology-controlled silicas in versatile applications of industrial, economical, medical, and environmental significance have also been discussed here elaborately. The scope of this review is limited to silica and silica based materials only. Other porous materials say, oxides, oxide composites, carbon, porous polymers, etc., their synthesis strategies and applications are not included here.

Morphology-controlled synthesis of silica and silica-based materials is quite challenging and well-defined since the method of preparation highly dependent on the reaction conditions and the materials used in the whole procedure. Soft templating approach using cationic, anionic non-ionic, and mixture of two or more surfactants have been used to synthesize these silicas in sol-gel media, followed by ageing for definite time under hydrothermal conditions or room temperature. For hollow sphere, yolk-shell, or core-shell silica, hard-templating methodology is adopted to prepare the solid sphere around which mesoporous shell is developed while using soft-templating pathway. However, a more in-depth investigation is necessary about the synthesis mechanisms of theses silica nanoparticles and a huge number of scientists all over the world are already involved in this research.

Recently, more emphasis is given to the functions and applications of these morphology oriented silica nanospheres. Silica nanospheres applied in catalysis, energy storage, biosensing, adsorption, and biomedical purposes have been discussed in this review. Although, the application of hollow, core-shell, or yolk-shell silica in different applied field is still at an early stage and more focus should be given on the catalytic, adsorption, and energy applications of these materials. However, we believe that our review work will be a guiding platform for those scientists who want to work on the morphology based nanoparticle synthesis and its applications in emerging fields of studies.

## List of Abbreviations

MCM-41	Mobil Composition of Matter-41
BET	Brunauer–Emmett–Teller
SDA	Structure directing agents
PMOs	Periodic mesoporous silicas
pH	-log[H^+^]
CMC	Critical miceller concentration
nm	Nanometer
CTAB	Cetyltrimethylammonium bromide
IUPAC	International Union of Pure and Applied Chemistry
TEOS	Tetraethyl orthosilicate
EtOH	Ethanol
C_16_TMACl	Cetyltrimethylammonium chloride
CPB	Cetylpyridinium bromide
3D	Three dimensional
TEA	Triethanolamine
TBOS	Tetrabutyl orthosilicate
HMS	Hollow mesoporous silica
PS	Polystyrene
SBF	Simulated body fluid
PMAA	Poly(methacrylic acid)
DSSC	Dye-sensitized solar cell
MSN	Mesoporous silica nanoparticles
EtOAc	Ethyl acetate
BTDS	bis [3-(triethoxysilyl)propyl] disulphide
APTES	3-aminopropyltriethoxysilane
RB	Rhodamine B
µM	Micromolar
FC	Fluorocarbon
TMOS	Tetramethyl orthosilicate
GO	Graphene oxide
